# Supplementary material for: Factors influencing medical students’ decisions to remain at their alma mater for postgraduate clinical training: A cross-sectional study in Japan
Source: PLoS One. 2025 Jul 10;20(7):e0327502. doi: 10.1371/journal.pone.0327502 (PMC12244655; doi:10.1371/journal.pone.0327502)
Supplement: Attachment — Submitted filename: Reviewers comments.pdf [file pone.0327502.s003.pdf]

Thank you for the opportunity to review the article entitled, “Factors Influencing Medical Students' Decisions to Remain at Their Alma Mater for Residency Training: A Cross-sectional Study in Japan.” This study aimed to investigate how faculty and hospital characteristics influence medical graduates' decisions to apply to their alma mater for postgraduate clinical training in Japan. The authors have collected data from different datasets and websites and chosen the related variables to determine the key factors that influence medical students’ decisions.
Although the manuscript is thoughtfully written and contributes significantly to university hospitals attracting medical applicants for PGY training, I believe it could benefit from addressing the points outlined below to further enhance its clarity and impact. I hope my comments will be helpful.

Title:

1. While the title fits well with the manuscript, I suggest using consistent terminology for "postgraduate training" throughout the document.

Abstract:

1. L.35. I think “Multivariate linear regression” is a misuse term in the abstract. Due to you have only one outcome variable (the number of medical students applying to their alma mater for postgraduate training), “multivariable linear regression” or “multiple regression” is more suitable.
2. L.31-33. In the abstract, you mentioned that this study would use the variable, the number of medical students applying to their alma mater for postgraduate training. However, in the Data analysis section, you mention that you used “the likelihood of medical…. (L.171-172)” Please make it clear.

Introduction:

1. L.74-75. “the 2022 data from the JRMP……63.5% of trainees 75 opting for community hospitals, compared with only 36.5% choosing university settings.” Is this a special phenomenon being Japan or do other countries also mention this issue? For example, in the United States and Australia, which you had mentioned in the Introduction (L.60).
2. L.84-87. Why you want to add the role of faculty characteristics as independents variables. Any reference support?

Materials and Methods:

1. What is the time period or year of the data you retrieved?
2. What do you mean by “university hospital”?

3. The terms "Rate" and "Ratio" have different statistical meanings. Please clarify this distinction and use the correct term.
4. L137-140. Why is the denominator of "the rate of medical students applying to their alma mater for postgraduate clinical training" "recruitment capacity specific to each facility" instead of the number of medical students in that year (the academic year of 2022)?
5. L.150-153. Does "Duration of medical practice" refer to "length of service/seniority"? How could you ensure that physician professors started working in the hospital immediately after they graduated?
6. Given that the quality of training and patient diversity may influence medical students' decisions (L.78-79); did you gather this data? I recommend treating these factors as confounding variables in data analysis.
7. Please clarify the term "multivariate linear regression" or "multivariable linear regression/multiple regression."
8. L.167-168. You mentioned "retention of medical school graduates at alma mater hospitals," did you mean retention rate or application rate? It is a little bit confusing. I would suggest to use consistent term.
9. Please clarify and re-organize the data analysis section (L.168-176). Why you only analyzed three variables in the univariate linear regression? How did you define potential confounders? What were your independent variables (factors) in the multiple regression?

Results:

1. What was the difference between "Percentage of medical students applying to their alma mater for postgraduate clinical training (%)" and "Percentage of medical students applying to their alma mater for postgraduate clinical training/enrollment capacity (%)" in Table 1?
2. The data of "Average daily outpatient visits (1000 patients)" was missing in Table 1.
3. "recruitment quota of the university hospital (L.198)" and "recruitment capacity specific to each facility (L.140)"? Please use consistent wording.
4. For the univariate analysis, salary showed no significant relationship with the application rate. Therefore, the statement, "There was also a trend of higher salaries …for postgraduate clinical training ($p = 0.0929$)," may not be appropriate.
5. For the footnote of Table 2, some of the adjust variables were not significant related to the outcome variables (the application rate). Please explain the adjust variables for multivariate analysis in the Result section.

6. Please clarify the outcome variable is retention rate or application rate in content and Table 2.
7. You mentioned that you would collect the average daily outpatient visits and average daily inpatient admission, but those variables did not show in the analysis (Table 2). Additionally, you have two new variables (number of departments and number of discharge patients) in Table 2, which were not mentioned in the methods section.
8. L.222. delete "Furthermore."

Discussion:

1. L.237-240. In my understanding, all (N=81) of your included hospitals were at the same level—university hospital. You just compare their location (urban or rural). However, Nishizaki et al. [18] compared community hospitals and university hospitals, which compared different levels of hospital. Or is the community hospitals were all in rural and the university hospitals were all in urban in Nishizaki et al.'s study?
2. L.244-248. The linkage between 6-year medical training and the institution's concerns is unclear.
3. L.258-260. It is unclear that the correlation of university recruitment culture and your finding. Please explain it more.
4. The limitation section is comprehensive. However, I would suggest providing some solutions for each limitation for future study.

Conclusion:

1. Conclusion is well fit with the study content.

References:

1. Please recheck your reference list. Some of institution names are unclear.
2. Most of the references you use in the discussion section are old documents (10 years ago) or in Japanese; please update the cited documents to new ones and use the English references to help foreign readers understand

Other:

The writing was unclear in some areas making it difficult to follow. The authors may benefit from support with editing.
