# Supplementary material for: Factors influencing medical students’ decisions to remain at their alma mater for postgraduate clinical training: A cross-sectional study in Japan
Source: PLoS One. 2025 Jul 10;20(7):e0327502. doi: 10.1371/journal.pone.0327502 (PMC12244655; doi:10.1371/journal.pone.0327502)
Supplement: Attachment — Submitted filename: Response_to_reviewers.docx [file pone.0327502.s005.docx]

**Responses to the Reviewers’ Comments**

Thank you for giving us the opportunity to submit a revised draft of our manuscript, titled “Factors Influencing Medical Students' Decisions to Remain at Their Alma Mater for Postgraduate Clinical Training: A Cross-sectional Study in Japan” for publication in *PLOS ONE*. We appreciate the time and effort that you and the reviewers dedicated to providing feedback on our manuscript, and we are grateful for the insightful comments. We have incorporated most of the suggestions made by the reviewers. These changes are highlighted within the manuscript for your convenience. Additionally, we have provided point-by-point responses to the reviewers’ comments below in blue. All page and line numbers refer to the revised manuscript file with tracked changes.

Comments from the Editorial Office:

**Comment 1:** Please ensure that your manuscript meets *PLOS ONE*'s style requirements, including those for file naming. The PLOS ONE style templates can be found at

<https://journals.plos.org/plosone/s/file?id=wjVg/PLOSOne_formatting_sample_main_body.pdf> and

<https://journals.plos.org/plosone/s/file?id=ba62/PLOSOne_formatting_sample_title_authors_affiliations.pdf>

**Author response:** We are grateful to the Editorial Office for the critical comments and helpful suggestions that have helped us improve our paper considerably. As indicated in the responses that follow, we have considered all these comments and suggestions in the revised version of our paper (text highlighted in pink).

**Comment 2:** We note that there is identifying data in the Supporting Information file <Data.xlsx>. Due to the inclusion of these potentially identifying data, we have removed this file from your file inventory.

Please remove or anonymize all personal information, ensure that the data shared are in accordance with participant consent, and re-upload a fully anonymized data set.

**Author Response:**

Thank you for your comment regarding data anonymization. We confirm that the dataset used in this study was compiled exclusively from publicly available sources, including university hospital websites, national databases, and official matching program results. However, we acknowledge that even publicly available information, such as program ID numbers, could potentially be used in combination with other variables to reidentify specific institutions. To fully comply with the data policy of *PLOS ONE* and safeguard against any possibility of indirect identification, we have removed the program ID column from the revised dataset. The updated Supporting Information file (Anonymized_Data.xlsx) has been re-uploaded and includes no direct or indirect identifiers.

**Comment 3:** Please review your reference list to ensure that it is complete and correct. If you have cited papers that have been retracted, please include the rationale for doing so in the manuscript text, or remove these references and replace them with relevant current references. Any changes to the reference list should be mentioned in the rebuttal letter that accompanies your revised manuscript. If you need to cite a retracted article, indicate the article’s retracted status in the References list and also include a citation and full reference for the retraction notice.

**Author Response:**

We have thoroughly reviewed our reference list to ensure its completeness and accuracy. No retracted papers were identified among the cited references. Additionally, we have updated and standardized the reference formatting in accordance with the style guidelines of *PLOS ONE*. These changes have been incorporated into the revised manuscript (pages 20–23, lines 441–511).

**Comment 4:** Can you please complete Table 1, the columns after average out patient visits (in 1000's) has been left blank.

kindly provide this information.

**Author Response:**

Thank you for your comment regarding Table 1. In the process of revising the manuscript for improved accuracy, we have updated our data source to the DPC dataset, which is a nationwide database focused on inpatient care in Japan. Because this dataset does not include outpatient statistics and its structure differs from other publicly available sources, we initially excluded outpatient visit data from the table. However, in response to your comment, we have added the average daily outpatient visits as a supplementary reference, using data obtained from the national residency information website “ResiNavi.” These values are presented in units of 1000 patients per day. A corresponding footnote has been added to Table 1 to clarify the data source and contextualize its interpretation.

Specific changes:

Table 1 (pages 10–11): We added the following row under continuous variables:

Average daily outpatient visits (×1000 patients): Mean = 1.79, SD = 0.69

Footnote to Table 1 (page 11, line 228–230):

“Average daily outpatient visits were obtained from the ResiNavi website and are reported in units of 1000 patients per day. These values were included for reference only, as reporting for-mats vary across institutions.”

**Comment 5:** in the discussion kindly remove lines 244 - 248 they are interrupting the discussion of the results.

**Author Response:**

Thank you for your suggestion. We agree that the text on those lines interrupt the flow of the Discussion. Accordingly, we have removed this portion from the revised manuscript to improve clarity and coherence in presenting our findings.

**Comment 6:** Kindly provide a short summary of the results in the beginning of the discussion

**Author Response:**

Thank you for your helpful suggestion. To improve the clarity and flow of the Discussion section, we have revised the opening paragraph to include a concise summary of the main results. This provides a clearer starting point for interpreting our findings in the broader context of previous research.

Specific change:

Page 13, lines 264–271

“This study examined how institutional characteristics influence medical students’ decisions to remain at their alma mater for postgraduate clinical training. Using data from 80 university hospitals in Japan, we conducted multiple linear regression analysis to identify key fac-tors associated with students’ application behavior. Students were more likely to choose their alma mater when (1) the proportion of alumni professors was lower and (2) the university hospital was located in a rural area. These findings suggest that institutional culture and geographic setting may shape medical students’ residency choices, even among hospitals of the same aca-demic level.”

**Comment 7:** and discuss why the presence of alumni professors could interfere with the choice of residency, as authors you have chosen this as a important factor to analyse, it would be useful for the readers to understand

why and how you had thought of this factor and why you think it may impact residency recruitment?

**Author Response:**

Thank you for this valuable comment. We agree that the rationale for including the proportion of alumni professors as a key factor should be more clearly articulated. In Japan, some university hospitals are perceived to have entrenched academic networks dominated by faculty who are alumni of the institution, and this has raised concerns that institutional cultures may become insular, potentially limiting exposure to diverse ideas and perspectives. We hypothesized that such academic homogeneity could influence medical students’ perceptions of the learning environment and mentorship opportunities, thereby affecting their decision to remain at their alma mater for postgraduate training. To clarify this point, we have revised the Discussion section to briefly explain our reasoning behind including this variable.

Specific changes:

Pages 15–16, lines 318–327

“We considered the proportion of alumni professors to be a potential indicator of institutional culture. University hospitals with a high percentage of faculty members who are also alumni may be perceived as having more homogeneous academic environments. In Japan, this pattern often reflects the traditional “Ikyoku” system (an in-house faculty recruitment structure unique to Japanese medical schools), in which universities tend to recruit their own graduates into faculty positions. Such an inward-looking recruitment practice may signal a closed institutional culture, potentially limiting students’ exposure to alternative perspectives or educational approaches. This could influence how medical students assess the openness of their training environment, making them more likely to seek opportunities outside their alma mater.”

**Comment 8:** Kindly address all the comments provided by the reviewers.

**Author Response:**

We have addressed all the comments provided by the reviewers.

Although this point was not raised by the reviewers, we recognized that the text in the Hospital selection (page 6, lines 120–130) and Proportion of medical students applying to their alma mater for postgraduate clinical training sections (page 7, lines 147–159) might lead to confusion regarding the inclusion and exclusion of certain institutions. Thus, to improve clarity and consistency for international readers, we have added brief explanations in both sections to state that the “National Defense Medical College and Jichi Medical University were excluded owing to institutional constraints that prevent graduates from participating in the general residency matching system.” This addition was made solely to enhance transparency and does not affect the study design or results.

Revised text (Page 6, lines 128–130), (page 7, lines 157–159):

The National Defense Medical College and Jichi Medical University were excluded owing to institutional constraints that prevent graduates from participating in the general residency matching system.

Further, to enhance clarity and maintain consistency across the manuscript, we have revised several variable labels in Table 1 (page 10-11). These edits ensure alignment with the definitions provided in the Variables and Data Analysis sections. Specifically, we have standardized terminology (e.g., “Years of medical practice for professors”) and clarified measurement units (e.g., “Average daily outpatient visits (per 1,000 patients)”) to avoid ambiguity. These changes were purely editorial and did not affect any statistical results or interpretations.

Revised text (Table 1, Page 10–11):

Years of medical practice for professors

Average daily outpatient visits (per 1,000 patients)

Number of clinical departments

Annual salary for residents (million yen)

Reviewer #1:

**Comment 1:** Thank you for the opportunity to review the article entitled, “Factors Influencing Medical Students' Decisions to Remain at Their Alma Mater for Residency Training: A Cross-sectional Study in Japan.” This study aimed to investigate how faculty and hospital characteristics influence medical graduates' decisions to apply to their alma mater for postgraduate clinical training in Japan. The authors have collected data from different datasets and websites and chosen the related variables to determine the key factors that influence medical students’ decisions.

Although the manuscript is thoughtfully written and contributes significantly to university hospitals attracting medical applicants for PGY training, I believe it could benefit from addressing the points outlined below to further enhance its clarity and impact. I hope my comments will be helpful.

**Author Response:** We appreciate your critical comments and useful suggestions that have helped us improve our paper considerably. As indicated in the responses that follow, we have considered all these comments and suggestions in the revised version of our paper (text highlighted in yellow).

Title:

**Comment 2:** While the title fits well with the manuscript, I suggest using consistent terminology for "postgraduate training" throughout the document.

**Author Response:**

Thank you for pointing out the inconsistency in terminology. In response to your suggestion and to ensure clarity, we have standardized the terminology throughout the manuscript. Specifically, we have changes various expressions such as “residency training,” “postgraduate training,” and “postgraduate education” to “postgraduate clinical training.” This term was selected because it aligns not only with your recommendation for consistent usage, but also with the official terminology used by the MHLW to describe the national 2-year training system for medical graduates.

A few representative changes are as follows:

- “postgraduate training” → “postgraduate clinical training”
- “residency training” → “postgraduate clinical training”
- “residency programs” → “postgraduate clinical training programs”

These revisions have been applied consistently throughout the Abstract, Introduction, Results, and Discussion sections.

**Abstract:**

**Comment 3:** L.35. I think “Multivariate linear regression” is a misuse term in the abstract. Due to you have only one outcome variable (the number of medical students applying to their alma mater for postgraduate training), “multivariable linear regression” or “multiple regression” is more suitable.

**Author Response:**

Thank you for pointing out the need for accurate terminology regarding the use of “multivariate.” To better reflect the nature of our statistical analysis—where a single dependent variable was modeled with multiple independent variables—we have replaced all instances of “multivariate” with “multiple regression.” This revision ensures clarity and consistency throughout the manuscript.

The following changes were made:

Page 2, lines 36: “multivariate linear regression analyses” to “multiple linear regression analyses”

Page 2, line 39: “the multivariate analysis” to “multiple regression analysis”

Page 10, lines 210: “a multivariate regression model” to “the multiple regression model”

Table 2. Title: “multivariate” to “multiple regression analyses”

Table 2, Column label: “Multivariate” to “Multiple regression”

Table 2. Note: “The multivariate analysis included...” to “The multiple regression analysis included...”

Page 13, lines 254: “results of multivariate analysis” to “results of multiple regression analysis”

**Comment 4:** L.31-33. In the abstract, you mentioned that this study would use the variable, the number of medical students applying to their alma mater for postgraduate training. However, in the Data analysis section, you mention that you used “the likelihood of medical…. (L.171-172)” Please make it clear.

**Author Response:**

Thank you for pointing this out. We agree that the terms “likelihood” and “number” were inconsistent with the actual variable used in the analysis. To avoid confusion and maintain consistency in terminology, we have replaced “likelihood” and “number” with “proportion” in the relevant sections of the manuscript. These modifications were made throughout the manuscript, including in the Abstract, Methods, and Results sections, to ensure consistent use of the term “proportion.”

Introduction:

**Comment 5:** L.74-75. “the 2022 data from the JRMP……63.5% of trainees 75 opting for community hospitals, compared with only 36.5% choosing university settings.” Is this a special phenomenon being Japan or do other countries also mention this issue? For example, in the United States and Australia, which you had mentioned in the Introduction (L.60).

**Author Response:**

Thank you for this insightful question. We have clarified that this trend is specific to Japan. Since the 2004 reform of the postgraduate training system, many Japanese medical graduates have preferred community hospitals over university hospitals for residency training owing to factors such as better salaries, work-life balance, and greater hands-on opportunities (Koike et al., 2010; Enari & Hashimoto, 2013). Community hospitals are often perceived as offering stronger clinical exposure, particularly in common diseases, which is supported by higher GM-ITE scores among their residents (Nishizaki et al., 2021). By contrast, in countries such as the United States and Australia, university-affiliated hospitals remain the primary sites for residency training and are valued for their academic structure and prestige (Heist & Torok, 2019; ACGME, 2024). We are not aware of a similar national trend in these countries. To address this point, we have added clarification in the Discussion section.

Specific changes:

Page 14, lines 278–284:

“While this shift has been well documented in Japan, similar nationwide preferences for com-munity hospitals over university hospitals have not been widely reported in countries such as the United States or Australia. In these countries, university-affiliated teaching hospitals remain the primary sites for postgraduate clinical training owing to their structured educational frameworks and academic prestige [17]. This contrast underscores the unique institutional and cultural context shaping Japan’s residency landscape.”

**Comment 6:** L.84-87. Why you want to add the role of faculty characteristics as independents variables. Any reference support?

**Author Response:**

Thank you for your comment. The inclusion of faculty characteristics (e.g., proportion of alumni professors and academic productivity) as explanatory variables was based on expert discussions and prior studies conducted before or during the design phase of our study (2022–2023). For instance, Nishizaki et al. (2021) reported that residents at university hospitals tended to score lower in clinical domains, suggesting differences in educational culture and faculty engagement. Watanabe et al. (2023) also reported that structural characteristics of university hospitals were associated with program popularity, which is potentially influenced by the presence and profile of faculty members. These findings provided a rationale for examining how faculty-related factors might be associated with medical students' application behavior.

Specific change:

Page 4, lines 90–93:

“ We included faculty-related variables such as the proportion of alumni professors and academic productivity, based on previous studies reporting associations between hospital educational environments and residency outcomes [7, 12].”

**Materials and Methods:**

**Comment 7:** What is the time period or year of the data you retrieved?

**Author Response:**

Thank you for your comment. We confirm that all data used in this study were retrieved from publicly available sources for the 2022 academic year, unless stated otherwise. We have added this information to the Methods section to clarify the time frame of data collection.

Specific change:

Page 5, lines 110–111:

“All data were collected from publicly available sources for the 2022 academic year in Japan, unless stated otherwise.”

**Comment 8:** What do you mean by “university hospital”?

**Author Response:**

Thank you for your comment. We understand that the term “university hospital” may not be immediately clear to international readers. To address this point, we have added a clarifying sentence in the Hospital selection section, describing their role as academic medical centers affiliated with medical schools in Japan. This is consistent with national-level descriptions of Japanese medical education, where university hospitals are identified as the primary sites for clinical clerkships and postgraduate training (Nishigori, 2024).

Specific change:

Page 5, lines 122–124:

“These hospitals function as academic medical centers affiliated with medical schools and serve as designated sites for postgraduate clinical training in Japan. They are commonly referred to as “university hospitals” in Japan [5].”

**Comment 9:** The terms "Rate" and "Ratio" have different statistical meanings. Please clarify this distinction and use the correct term.

**Author Response:**

Thank you for your comment. We agree that the term “rate” was inappropriately used in several instances where “proportion” or “percentage” would have been more accurate. Accordingly, we have carefully reviewed and revised the manuscript to ensure consistent and appropriate use of terminology. All instances of “rate” referring to unitless ratios were replaced with “proportion” or “percentage” (e.g., “proportion of medical students applying…”). However, the term “rate” was retained only when referring to time-based measures, such as “turnover rate.” These modifications were made throughout the manuscript, including in the Abstract, Methods, and Results sections, to ensure consistency.

**Comment 10:** L137-140. Why is the denominator of "the rate of medical students applying to their alma mater for postgraduate clinical training" "recruitment capacity specific to each facility" instead of the number of medical students in that year (the academic year of 2022)?

**Author Response:**

Thank you for your comment. We understand your concern regarding the use of “recruitment quota” as the denominator in calculating the proportion of medical students applying to their alma mater for postgraduate clinical training. In the Japanese postgraduate clinical training system, residency placement is determined through a national matching process that is not restricted to graduates of a given institution. Therefore, the number of graduates at a medical school does not necessarily reflect the number of training positions at its affiliated university hospital.

Moreover, recruitment quotas vary across hospitals, and some facilities do not fill all their positions in the matching process. Therefore, using the number of graduates as the denominator would introduce inconsistencies and obscure the actual competitiveness or popularity of each hospital.

To accurately measure institutional popularity or retention capacity, we used the recruitment quota, i.e., the number of training positions officially allocated to each university hospital, as the denominator. This allows for a standardized comparison across institutions regardless of the number of graduates. This approach is consistent with national reporting standards (Nishigori, 2024). We have added a brief explanation in the Methods section to clarify this point.

Specific change:

Page 7, lines 154–155:

“Since the number of graduates and the number of training positions do not always match at each hospital, using the recruitment quota as the denominator allows for a fair comparison of institutional popularity [5].”

**Comment 11:** L.150-153. Does "Duration of medical practice" refer to "length of service/seniority"? How could you ensure that physician professors started working in the hospital immediately after they graduated?

**Author Response:**

Thank you for your insightful comment. In this study, “years of medical practice” refers to the number of years since each professor obtained their medical degree. We estimated this by subtracting their graduation year from 2022.

While this may not capture the exact timing of when they began clinical work in Japan, medical graduates universally begin postgraduate training immediately after graduation. Extended delays or career changes are extremely rare, particularly among those who become professors later. Therefore, this approach provides a practical and standardized estimate of their clinical career length. To improve clarity, we have replaced the phrase “duration of medical practice” with “years of medical practice” throughout the manuscript.

Specific change:

Page 8, lines 174–176:

“This served as a proxy for their clinical career length, as physicians in Japan typically begin postgraduate training immediately after graduation; extended gaps are rare, particularly among those who attain professorships later.”

**Comment 12:** Given that the quality of training and patient diversity may influence medical students’ decisions (L.78-79); did you gather this data? I recommend treating these factors as confounding variables in data analysis.

**Author Response:**

Thank you for this valuable comment. As noted in the Introduction (lines 80–82), we referred to previous studies highlighting training quality and the variety of patient cases as important factors influencing medical students’ decisions. We agree that these variables may act as potential confounders. However, as publicly available, standardized data on these variables are currently lacking across institutions in Japan, we were unable to include them in our regression models. We have now acknowledged this limitation explicitly in the revised manuscript.

We have also added a note on the potential to address this gap in future studies using institutional surveys or national-level clinical databases, such as the NDB.

Specific change:

Page 20, lines 413–417:

"Lastly, although previous studies suggest that the quality of training and the variety of patient cases are important determinants of hospital choice, such data are not uniformly available from public sources and were therefore not included in the present analysis. Future research should address this limitation by incorporating institution-level surveys or clinical datasets.”

**Comment 13:** Please clarify the term “multivariate linear regression” or “multivariable linear regression/multiple regression.”

**Author Response:**

Thank you for this important comment. As noted in a similar point raised earlier, we have reviewed and corrected all instances of “multivariate” throughout the manuscript to ensure terminological accuracy. Specifically, we have replaced “multivariate linear regression” and “multivariate analysis” with “multiple linear regression” for consistency. These corrections have been applied consistently in the Abstract, Methods, Results, and Table 2.

**Comment 14:** L.167-168. You mentioned “retention of medical school graduates at alma mater hospitals,” did you mean retention rate or application rate? It is a little bit confusing. I would suggest to use consistent term.

**Author Response:**

Thank you for your comment. We agree that the term “retention” may be misleading in this context. Since our analysis focused on students who applied to their alma mater hospitals—not those who enrolled—we have replaced “retention” with “proportion of students applying to their alma mater” throughout the manuscript for clarity and consistency.

Specific changes:

Page 2, lines 36–37:

“to assess factors associated with graduate retention in alma mater hospitals.”

→ “to assess factors associated with the proportion of medical students applying to their alma mater hospitals.”

Page 9, lines 205–210:

“Univariate and multiple linear regression analyses were performed to evaluate factors associated with the retention of medical school graduates at alma mater hospitals. Initially, a univariate analysis was used to assess the impact of several variables, including the percentage of alumni professors, number of beds, and number of discharged patients, on graduate retention.”

→ “Statistical analyses were conducted to examine the association between institutional fac-tors and the proportion of medical students applying to their alma mater for postgraduate clinical training. Univariate linear regression analyses were first performed for each explanatory variable described in the variables section. Variables with a p-value < 0.05 in univariate models or those considered theoretically relevant were included in the multiple regression model.”

Page 12, Table 2 Title:

“retention of medical school graduates at”

→ “the proportion of medical students applying to”

Page 12, Table 2 Note:

“retention rate of medical school graduates within the same institution as the resident physicians.”

→ “proportion of medical students applying to their alma mater hospitals for postgraduate clinical training.”

Page 16, lines 327–328:

“retention of medical graduates”

→ “the proportion of medical students applying to their alma mater hospitals”

**Comment 15:** Please clarify and re-organize the data analysis section (L.168-176). Why you only analyzed three variables in the univariate linear regression? How did you define potential confounders? What were your independent variables (factors) in the multiple regression?

**Author Response:**

Thank you for this important comment. We agree that our original Data analysis section lacked clarity regarding the selection and handling of variables. To improve transparency, we have revised the structure of the Methods section by (1) adding a dedicated “Variables” subsection that clearly defines all explanatory and outcome variables used in the analysis and (2) rewriting the Data analysis paragraph to explicitly describe our modeling strategy, including how potential confounders were identified and selected for multivariable regression.

Specific changes:

Pages 7–8, lines 185–201: Added a new subsection titled Variables, which defines each explanatory variable and its source.

“Variables

The outcome variable was the proportion of medical students applying to their alma mater for postgraduate clinical training, calculated as the number of applicants from the same university divided by the recruitment quota for each university hospital.

We considered the following explanatory variables based on prior literature and expert con-sultation: percentage of alumni professors, calculated from publicly available faculty data; years of medical practice for professors, defined as the number of years since graduation from medical school (i.e., 2022 minus graduation year); number of hospital beds, extracted from official hospital webpages; number of clinical departments, counted from hospital department listings; average daily number of discharged patients, derived from the national DPC Data-base and scaled per 100 patients; average daily outpatient visits, obtained from the ResiNavi website and scaled per 1000 patients; presence of female professors, categorized as none, one, or two or more; publication in top five medical journals, binary indicator based on faculty publication records; number of affiliated hospitals, categorized as none, one, or two or more; urban or rural location, based on geographic classification (government-designated cities or 23 wards in Tokyo); and type of university, classified as national/public versus private; salary for residents, annual salary (in 10 000 yen), excluding bonuses and housing allowance.”

Page 9, Lines 205–213: “ Statistical analyses were conducted to examine the association between institutional fac-tors and the proportion of medical students applying to their alma mater for postgraduate clinical training. Univariate linear regression analyses were first performed for each explanatory variable described in the variables section. Variables with a p-value < 0.05 in univariate models or those considered theoretically relevant were included in the multiple regression model. Multicollinearity was checked before model fitting. Regression coefficients, 95% confidence intervals (CIs), and p-values are reported. A p-value < 0.05 was considered statistically significant, and all statistical analyses were performed using SAS (version 9.4; SAS Institute Inc., Cary, NC, USA).”

**Results:**

**Comment 16:** What was the difference between “Percentage of medical students applying to their alma mater for postgraduate clinical training (%)” and “Percentage of medical students applying to their alma mater for postgraduate clinical training/enrollment capacity (%)” in Table 1?

**Author Response:**

Thank you for this helpful comment. We agree that the distinction between these two variables was not sufficiently clear. The two variables differ in their definitions and purposes. “Proportion of medical students applying to their alma mater for postgraduate clinical training / enrollment capacity (%)” reflect the relative popularity of each university hospital by indicating how many medical students from that university applied to return for residency, standardized by the number of training positions available. Conversely, “percentage of matched residents who are graduates of the same university (%)” reflect the composition of the actual matched residents and indicates how many of those who started residency at each hospital were alumni.

Since our analysis focused on the former variable, which measures the popularity of each institution, and the latter variable was not used in any analysis, we have removed it from Table 1 to avoid potential confusion, particularly for international readers less familiar with Japan's residency matching system.

Specific change:

Table 1: Removed the row titled “Percentage of matched residents who are graduates of the same university (%)”

**Comment 17:** The data of “Average daily outpatient visits (1000 patients)” was missing in Table 1.

**Author Response:**

Thank you for your comment regarding Table 1. In the process of revising the manuscript for greater accuracy, we have updated our data source to the DPC dataset, which is a nationwide database focused on inpatient care in Japan. Because this dataset does not include outpatient statistics and its structure differs from other publicly available sources, we initially excluded outpatient visit data from the table. However, in response to your comment, we have added the average daily outpatient visits as a supplementary reference, using data obtained from the national residency information website “Resinavi.” These values are presented in units of 1000 patients per day. A corresponding footnote has been added to Table 1 to clarify the data source and contextualize its interpretation.

Specific changes:

Table 1: We added the following row under continuous variables:

Average daily outpatient visits (×1000 patients): Mean = 1.79, SD = 0.69

Footnote to Table 1 (page 11, lines 228–230):

“Average daily outpatient visits were obtained from the ResiNavi website and are reported in units of 1000 patients per day. These values were included for reference only, as reporting for-mats vary across institutions.”

**Comment 18:** “recruitment quota of the university hospital (L.198)” and “recruitment capacity specific to each facility (L.140)”? Please use consistent wording.

**Author Response:**

Thank you for your valuable comment. We agree that the terms “recruitment quota” and “recruitment capacity” were inconsistently used to refer to the same concept. To improve clarity and consistency, we have used “recruitment quota” consistently throughout the manuscript, which accurately reflects the official number of residency slots offered by each university hospital.

Specific change:

Page 11, lines 227:

Replaced “recruitment capacity specific to each facility” with “recruitment quota of the university hospital”

Page 11, Table 1 Note:

“Percentage of medical students applying to their alma mater for postgraduate clinical training / enrollment capacity (%)”

→ “Percentage of medical students applying to their alma mater for postgraduate clinical training / recruitment quota (%)”

**Comment 19:** For the univariate analysis, salary showed no significant relationship with the application rate. Therefore, the statement, “There was also a trend of higher salaries …for postgraduate clinical training (p = 0.0929),” may not be appropriate.

**Author Response:**

Thank you for this valuable comment. We agree that describing a non-significant association (p = 0.0929) as a “trend” may be inappropriate and potentially misleading. Since this result was not discussed in the Discussion section and was not statistically significant, we have removed the corresponding sentence from the Results section to ensure clarity and precision in reporting.

**Comment 20:** For the footnote of Table 2, some of the adjust variables were not significant related to the outcome variables (the application rate). Please explain the adjust variables for multivariate analysis in the Result section.

**Author Response:**

Thank you for your comment. We agree that not all adjustment variables in the multiple regression model were significantly associated with the outcome. To clarify this point, we have added a new explanation in the Methods section that explicitly describes how the adjustment variables were selected. As stated there, the variables were chosen a priori based on expert opinion and relevant literature to account for potential confounding factors. Additionally, we have added a sentence in the Results section to reflect this rationale for including adjustment variables.

Specific change:

Pages 8–9, lines 185–201: As previously noted, we added a new subsection titled Variables, which defines each explanatory variable and its source

Page 9, line 211: Replaced the previous “Data analysis” paragraph with a revised version explaining the univariate screening, theoretical justification, and model building procedures

Revised text (Page 9, line 205–213):

“Statistical analyses were conducted to examine the association between institutional factors and the proportion of medical students applying to their alma mater for postgraduate clinical training. Univariate linear regression analyses were first performed for each explanatory variable described in the variables section. Variables with a p-value < 0.05 in univariate models or those considered theoretically relevant were included in the multiple regression model. Multicollinearity was checked before model fitting. Regression coefficients, 95% confidence intervals (CIs), and p-values are reported. A p-value < 0.05 was considered statistically significant, and all statistical analyses were performed using SAS (version 9.4; SAS Institute Inc., Cary, NC, USA).”

Page 13, line 258–261:

“Variables included in the multiple regression model were selected on the basis of their statistical significance in univariate analyses (p < .05) or theoretical relevance to ensure appropriate adjustment for potential confounding factors.”

**Comment 21:** Please clarify the outcome variable is retention rate or application rate in content and Table 2.

**Author Response:**

Thank you for your comment. As also addressed in Comment 14, we agree that the term “retention” was potentially misleading in this context. Since our outcome variable focused on students who applied to their alma mater hospitals, not those who ultimately enrolled, we have replaced all instances of “retention” with “proportion of medical students applying to their alma mater for postgraduate clinical training” throughout the manuscript to ensure clarity and consistency.

These revisions have been made in the Abstract, Methods, Results, Discussion, and Table 2.

Examples:

Page 2, lines 36–37, Abstract: “graduate retention” → “proportion of medical students applying to their alma mater hospitals”

Page 8, lines 186–187, Methods: “retention of medical school graduates at alma mater hospitals” → “proportion of medical students applying to their alma mater hospitals”

Table 2 title and footnote: Updated accordingly

**Comment 22:** You mentioned that you would collect the average daily outpatient visits and average daily inpatient admission, but those variables did not show in the analysis (Table 2). Additionally, you have two new variables (number of departments and number of discharge patients) in Table 2, which were not mentioned in the methods section.

**Author Response:**

Thank you for pointing this out. We acknowledge that the description of variables in the earlier version of the Methods section was incomplete and potentially misleading. We originally collected average daily outpatient visit data from the ResiNavi website to estimate hospital size. However, as noted in the revised Methods section, we ultimately used “average daily discharged patients” derived from the DPC database instead of “inpatient admissions,” as the DPC provides reliable and standardized data at the facility level. Although outpatient data were not used in regression analyses, we have retained this variable in Table 1 for descriptive purposes.

We decided not to use outpatient data in the analysis for the following reasons:

- The number of discharged patients, number of clinical departments, and number of beds together provide a more robust and objective measure of hospital size.
- Outpatient visit data are not standardized across institutions and may exhibit multicollinearity with inpatient volume measures (e.g., discharged patients).
- To ensure model stability and accuracy, outpatient data were excluded from univariate and multiple regression analyses.

We have revised the Methods section to more clearly reflect the final set of variables used in our analysis.

Specific changes:

Page 6, lines 134: “average daily outpatient visits, average daily inpatient admissions”

⟶ “average daily outpatient visits, average daily discharged patients”

Pages 8–9, lines 185–201: As noted previously, we added a new Variables subsection has been added to clarify all variables used in the study.

Page 10: In Table 1, the labels of the variables have been revised for consistency and clarity.

**Comment 23:** L.222. delete “Furthermore.”

**Author Response:**

Thank you for your comment. We agree that the word “Furthermore” at the beginning of that line was unnecessary. We have deleted this word from the revised manuscript to improve flow.

Discussion:

**Comment 24:** L.237-240. In my understanding, all (N=81) of your included hospitals were at the same level—university hospital. You just compare their location (urban or rural). However, Nishizaki et al. [18] compared community hospitals and university hospitals, which compared different levels of hospital. Or is the community hospitals were all in rural and the university hospitals were all in urban in Nishizaki et al.’s study?

**Author Response:**

Thank you for this insightful comment. As you correctly noted, our study differs from Nishizaki et al.’s study in that we focused exclusively on university hospitals and compared institutional characteristics within this group, particularly geographic setting (urban vs. rural). In contrast, Nishizaki et al. compared community hospitals with university hospitals, examining differences in institutional type.

To clarify the relationship between the two studies, we have revised the Discussion section to acknowledge that rural university hospitals in Japan may function similarly to community hospitals in terms of clinical experience and local healthcare roles. We believe that our findings build upon the work of Nishizaki et al. by showing that such characteristics—often associated with community hospitals—can also be observed within a subset of university hospitals. This internal variability helps explain why some university hospitals are more successful in retaining their graduates than others. We hope this clarification helps readers better understand our study within the broader research context of residency selection in Japan.

Specific changes:

Pages 13–14, lines 272-278:

“In Japan, university hospitals have traditionally been central to postgraduate training owing to their resources and specialization. However, many residents have recently shifted their preference toward community hospitals, which offer exposure to a broader range of common diseases and generalist experience. Prior research has also shown that residents in community hospitals outperform those in university settings on general clinical knowledge tests, e.g., the General Medicine In-Training Examination [12]. These differences may be driven by the volume and variety of clinical experiences in community settings.”

Page 14, lines 285–299:

“In this context, faculty composition may serve as a proxy for institutional culture. The observed negative correlation between the proportion of alumni professors and the proportion of students applying to their alma mater suggests that medical students may be more attracted to institutions that foster diversity in academic backgrounds. Universities with fewer alumni professors may be perceived as more open, inclusive, or meritocratic, thereby appealing to students seeking broader perspectives in their postgraduate training.

These findings build upon prior work by Nishizaki et al., who compared community and university hospitals and found structural and educational differences that influenced residency decisions. Although our study focused on university hospitals, our results demonstrate that institutional characteristics, i.e., rural location and faculty composition, can create environments within university hospitals that resemble those of community hospitals. Particularly, rural university hospitals may play a more regionally integrated role and offer broader clinical experiences than urban ones, which in turn may appeal to medical students seeking hands-on, generalist training. This internal variation within university hospitals helps to explain differential success in retaining graduates.”

**Comment 25:** L.244-248. The linkage between 6-year medical training and the institution’s concerns is unclear.

**Author Response:**

Thank you for your comment. We agree that the linkage between the 6-year medical training and institutional concerns was unclear. Upon further review and in consideration of the editor’s related suggestion, we have removed the related text to improve the clarity and coherence of the Discussion.

**Comment 26:** L.258-260. It is unclear that the correlation of university recruitment culture and your finding. Please explain it more.

**Author Response:**

Thank you for your helpful comment. We agree that the link between university recruitment culture and our findings merits clear explanation. To address this, we have expanded the Discussion section to explain how the proportion of alumni professors may serve as a proxy for institutional culture. Specifically, we discuss how universities with a lower proportion of alumni faculty may foster more open, inclusive, and meritocratic environments, which in turn may influence medical students' decisions to remain at their alma mater.

Specific changes:

Page 15–16, lines 318–327

“We considered the proportion of alumni professors to be a potential indicator of institutional culture. University hospitals with a high percentage of faculty members who are also alumni may be perceived as having more homogeneous academic environments. In Japan, this pattern often reflects the traditional “Ikyoku” system (an in-house faculty recruitment structure unique to Japanese medical schools), in which universities tend to recruit their own graduates into faculty positions. Such an inward-looking recruitment practice may signal a closed institutional culture, potentially limiting students’ exposure to alternative perspectives or educational approaches. This could influence how medical students assess the openness of their training environment, making them more likely to seek opportunities outside their alma mater.”

**Comment 27:** The limitation section is comprehensive. However, I would suggest providing some solutions for each limitation for future study.

**Author Response:**

Thank you for your valuable suggestion. We agree that proposing future directions can enhance our limitations section. We have revised the section to include possible solutions or future research directions for each limitation mentioned.

Additionally, to improve clarity and avoid redundancy, we removed the sentence, “For example, the impact of having a higher proportion of alumni professors on residency choices could be influenced by other unmeasured variables such as institutional culture,” as the preceding text already addressed the limitations in establishing causality and the potential influence of unmeasured confounders.

Specific change:

Page 18, lines 390–392:

“Future research should consider collecting data from a broader range of institutional and regional sources, within and outside Japan, to improve generalizability and enable international comparisons.”

Page 18, lines 395–396:

“To reduce potential confounding and better assess causal relationships, longitudinal or interventional study designs may be beneficial in future research.”

Page 19, lines 402–409:

“In Japan, these programs provide financial support (e.g., tuition waivers or living stipends) from local governments in exchange for a service obligation to practice within the sponsoring region after graduation. However, these programs are limited in scale, only a small number of students per prefecture participate, and not all prefectures offer such programs. Furthermore, even within these regions, the designated practice sites may vary in their rurality. Future studies should investigate how these obligations influence actual training site choices by collecting nationwide data on the placement patterns of regional quota graduates.”

Conclusion:

**Comment 28:** Conclusion is well fit with the study content.

**Author Response:**

Thank you for your positive comment. We are pleased to hear that you found the Conclusion section to be well aligned with the study content. No changes were made in response to this point.

References:

**Comment 29:** Please recheck your reference list. Some of institution names are unclear.

**Comment 30:** Most of the references you use in the discussion section are old documents (10 years ago) or in Japanese; please update the cited documents to new ones and use the English references to help foreign readers understand

**Author Response:**

Thank you for your valuable suggestions (Comments 29 and 30) regarding the reference list.

We have carefully reviewed the entire reference list and clarified the names of institutions to ensure consistency and transparency (pages 20–23, lines 441–511). Specifically, for web-based sources, we replaced ambiguous or generic author names with the appropriate organizational names (e.g., "Accreditation Council for Graduate Medical Education (ACGME)," "Australian Medical Council (AMC)," "Ministry of Health, Labour and Welfare (MHLW)," and "Mynavi Corporation") and formatted them accordingly. We have also removed duplicate mentions of the same institution name in the author and publisher fields. We also updated outdated links where necessary. For example, the previously cited Web of Science platform page was no longer available, so we revised the citation to reflect the current official portal maintained by Clarivate Analytics (https://webofsciencelearning.clarivate.com/learn), including updated access dates and accurate institutional authorship. Similarly, we added and formatted the citation for the ResiNavi website, specifying "Medical Principles Co., Ltd." as the author and including the access date and URL (https://www.residentnavi.com/).

Additionally, we have revised the Discussion section to include more recent and internationally accessible literature where appropriate. For example, we replaced or supplemented older Japanese-language references with more up-to-date and peer-reviewed English publications (e.g., Nishigori H. Medical education in Japan. Med Teach. 2024;46(sup1). [DOI: 10.1080/0142159x.2024.2372108]). This newer review offers comprehensive and accessible insights on Japan’s medical education and workforce context, which aligns with several themes discussed in our manuscript (e.g., residency system reforms, rural healthcare, and physician distribution). We have also ensured that key points supported by older government documents are supplemented with relevant English-language sources where available.

We hope these changes improve the accessibility and clarity of our reference list for international readers.

Other:

**Comment 31:** The writing was unclear in some areas making it difficult to follow. The authors may benefit from support with editing.

**Author Response:**

Thank you for your valuable feedback regarding the clarity of the text. In response, we have carefully revised the manuscript to improve readability and flow. Additionally, we sought support from a professional English editing service, and the revised manuscript has been edited accordingly. A certificate of English editing has been obtained and is available upon request.

Reviewer #2:

**Comment 1:** The article is interesting and well-written. From an international perspective, it reveals a reverse concern compared to many nations, in that it reveals a recruitment problem in urban areas in Japan. Most health care systems are challenged to recruit physicians to rural areas. The authors might well want to discuss there results with this international lens, focusing on likely causes of the disparity. The following issues need to be addressed:

**Author Response:** We appreciate your critical comments and valuable suggestions that have helped us improve our paper considerably. As indicated in the responses that follow, we have considered all these comments and suggestions in the revised version of our paper (text highlighted in blue).

**Comment 2:** The statistics of the article are well-performed. Where concerns arise regarding the data supporting conclusions, it may well be just be a matter of definition. The chief concern relates to the repeated assertion that recruitment could be helped by improved diversity of the faculty. Most readers would interpret improved diversity to mean race or gender. Race is not tested, and gender does not seem even close to being a significant contributor to recruitment. Instead, diversity seems to mean the proportion of faculty that are alumni. A better approach to describing this metric would remove confusion.

**Author Response:**

Thank you for pointing out the potential ambiguity in our use of the term “diverse,” which may suggest demographic diversity (e.g., race, gender) rather than institutional variation. To avoid misinterpretation and ensure consistency throughout the manuscript, we revised all instances of “diverse” that could be misleading. Specifically, we clarified that the diversity in question refers to faculty members’ institutional origins or professional backgrounds, in line with the main focus of our study.

The following changes were made accordingly:”

Specific changes for:

Page 2, line44:

” diverse faculty backgrounds” to “greater heterogeneity in faculty members’ institutional origins”

Page 3, line 46:

” fostering faculty diversity” to “recruiting faculty from a broader range of institutions”

Page 3, line 61:

” diverse medical environments” to “various clinical settings “

Page 4, line 81:

“patient diversity” to “the variety of patient cases”

Page 16, line 329:

“more diverse faculties” to “faculty drawn from a broader range of institutions”

Page 16, line 332:

“By contrast, a high proportion of alumni professors might create a perception of limited diversity in teaching and mentorship, which could make the institution less appealing to graduates seeking exposure to a broad range of professional perspectives.” To “By contrast, a high proportion of alumni professors might create a perception of institutional homogeneity or entrenched academic networks, potentially signaling a closed or in-group culture that discourages graduates seeking broader professional exposure.”

Page 16, line 336:

“to seek diverse experiences to benefit from various viewpoints outside their alma maters.” To “to look beyond their alma maters for training opportunities that provide a wider range of perspectives and are free from perceived academic in-groups.”

Page 16, line 346:

“diverse perspectives and experiences, making them receptive to guidance from professors who bring unique insights from their experiences at different institutions.” to “various perspectives and experiences, particularly those shaped by professors with different institutional backgrounds [22], making them receptive to guidance from such mentors.”

Page 20, line 425:

“diverse faculty backgrounds” to “faculty drawn from a broader range of institutions”

Page 16, line 346–348:

“various perspectives and experiences, particularly those shaped by professors with different institutional backgrounds [22], making them receptive to guidance from such mentors.”

**Comment 3:** The article includes some interesting data that should be discussed, such as the fraction of faculty that are female. At the moment this data seems to lack a denominator. expressed as a fraction, many researchers would find this useful additional information.

**Author Response:**

Thank you for this insightful suggestion. We agree that presenting the proportion of female professors would offer a more informative perspective. In our dataset of 80 university hospitals (excluding Jichi Medical University due to its unique faculty structure), we calculated the average proportion of female professors by dividing the number of female professors by the total number of professors at each institution. This analysis revealed that female professors comprised an average of 3.1% of all professors, with a standard deviation of 5.03. We have added this information to the descriptive table and clarified the calculation method in the Methods section.

Specific changes:

Table 1: Added “Number of professors, Number of female professors, Average proportion of female professors (%)”

Methods(Page 8): Added the following description:

Added “Proportion of female professors

The number of female and total professors at each university hospital was obtained from publicly available university websites. Based on these data, we calculated the proportion of female professors.

**Comment 4:** It would be useful to include a discussion of the factors that likely control the fraction of faculty that are alumni.

**Author Response:**

Thank you for your comment. We have revised the Discussion section to include a more explicit explanation of the factors that may influence the proportion of alumni professors. Specifically, we now refer to the traditional “Ikyoku” system in Japan, in which universities tend to recruit their own graduates into faculty positions. This inward-looking recruitment practice may shape institutional culture and help explain the observed association between faculty composition and students’ residency choices.

Specific changes:

Page 15, lines 318–327:

“We considered the proportion of alumni professors to be a potential indicator of institutional culture. University hospitals with a high percentage of faculty members who are also alumni may be perceived as having more homogeneous academic environments. In Japan, this pattern often reflects the traditional “Ikyoku” system (an in-house faculty recruitment structure unique to Japanese medical schools), in which universities tend to recruit their own graduates into faculty positions. Such an inward-looking recruitment practice may signal a closed institutional culture, potentially limiting students’ exposure to alternative perspectives or educational approaches. This could influence how medical students assess the openness of their training environment, making them more likely to seek opportunities outside their alma mater.”

Page 16, lines 337–340

“The proportion of alumni professors may reflect long-standing recruitment practices rooted in the Ikyoku system, where internal promotion and the hiring of institutional graduates have been traditionally favored.”

**Comment 5:** Much of the data does not seem easily available, particularly the data from websites of each university hospital, which are not specified or linked.

**Author Response:**

Thank you for your suggestion regarding the inclusion of university hospital URLs. In response, we have compiled a comprehensive list of the official English names and URLs of all the included university hospitals. This list has been added as “Supplemental File 1” to improve transparency and reproducibility. We have also incorporated a corresponding sentence at the end of the “University hospital information” section in the Methods section of the manuscript.

Specific change:

Page 6, lines 139–140:

“A list of official English names and URLs of the included university hospitals are provided in Supplemental File S1.”
